# Drug-Induced Sedation Endoscopy (DISE) DATA FUSION system: clinical feasibility study

**DOI:** 10.1007/s00405-017-4765-7

**Published:** 2017-10-17

**Authors:** Esuabom Dijemeni, Bhik Kotecha

**Affiliations:** 1 Department of Bioengineering, Imperial College London, London, UK; 2 Research and Development Department, DISE INNOVATION, London, UK; 3 ENT Department, Royal National Throat, Nose and Ear Hospital, 330 Grays Inn Road London, WC1X 8DA UK; 4 Barts and The London School of Medicine and Dentistry, London, E1 2AT UK

**Keywords:** Drug-induced sleep endoscopy, Data fusion, Upper airway obstruction, Obstructive sleep apnea, Data visualisation, DISE, Medical innovation, Medical device

## Abstract

Drug-induced sleep endoscopy (DISE) is a diagnostic technique for 3D dynamic anatomical visualisation of upper airway obstruction during sedated sleep. There is a lack of standardised procedure and objective measurement associated with information capture, information management, evaluation of DISE findings, treatment planning, and treatment outcomes. The objective of this study is to present clinical feasibility results using a DISE DATA FUSION system for capturing, merging, displaying and storing anatomical data from an endoscopic imaging system and cardiorespiratory data from an anaesthesiological monitoring system simultaneously in real-time during DISE. This prospective cohort study included 20 patients presenting with symptoms of sleep related breathing disorders undergoing drug-induced sedation endoscopy and had volunteered for DISE DATA FUSION system to be used during their DISE assessment. The DISE DATA FUSION system was used to capture, merge, display, and store anatomical changes from an endoscopic imaging system and cardiorespiratory changes from an anaesthesiological monitoring system simultaneously in real time during drug-induced sedation endoscopy assessment. In all 20 patients, anatomical obstructions at different levels of the pharyngeal lumen (soft palate, velum, tonsils, oropharynx lateral wall, base of tongue, and epiglottis) with a different obstruction configuration and severity were captured simultaneously in real time with its associated cardiorespiratory parameters. Furthermore, a composite video consisting of an anatomical image, blood oxygen level, pulse rate, blood pressure, and timestamp was created for every obstructive event. Our system provides a useful and better way of capturing, merging, visualising, and storing anatomical data/physiological data simultaneously during DISE in real time. Furthermore, it enhances the understanding of the impact of the anatomical severity due to the simultaneous display of the cardiovascular parameters at that specific time of anatomical obstruction for optimising surgical decision based on DISE.

**Electronic supplementary material:**

The online version of this article (doi:10.1007/s00405-017-4765-7) contains supplementary material, which is available to authorized users.

## Introduction

Drug-induced sleep endoscopy (DISE), also referred to as sleep nasendoscopy (SNE) and first proposed by Croft and Pringle, is a technique for direct visualisation of anatomical site or sites of obstruction in sleeping patients [1]. DISE is usually performed in the operating theatre with a patient under anaesthesia. A flexible endoscope is inserted through the nasal cavity, nasopharynx, oropharynx, and above larynx with the purpose of preventing irritation. Additional cardiorespiratory parameters (pulse rate, saturated blood oxygen level and blood pressure) are monitored. In some cases, a bispectral index (BIS) monitor may be used to monitor the depth of sedation during DISE. DISE is usually carried out by a multi-disciplinary team consisting of an otorhinolaryngologist, anesthesiologist and theatre nurses.

DISE has shown to:

1. Provide a 3D dynamic visualisation of the multi-segmental upper airway obstruction [2–4].
2. Provide a quality assessment of the dynamic upper airway event [5].
3. Provide useful information on patient management [6].
4. Improve treatment planning as compared to using the awake upper airway assessment technique [7].
5. Have a reliable intraobserver agreement [8].
6. Have moderate to substantial interrater reliability [9].
7. Have good test–retest reliability [10].
8. Have good agreement with polysomnography [11].

Some criticism of DISE includes:

1. Significant change in snoring patterns as compared to natural sleep and physiological sleep differs from sedation induced sleep [12, 13].
2. Upper airway obstruction pattern is dependent on experience [14].
3. Upper airway obstruction pattern is dependent on the sedation administration strategy [15].
4. Upper airway obstruction pattern is dependent on the administered dose [16].
5. Growing number of DISE classification systems [17].
6. Good analysis depends on experience [18].
7. 10–15 min snapshot evaluation of DISE compared to 6–8 h of natural sleep.
8. Stage of sleep during evaluation: REM vs non-REM sleep.
9. DISE is usually performed in supine position.

The non-anatomical clinical parameters monitored during DISE play an important role in understanding upper airway obstruction [18]. Pulse oximeter and/or anaesthesiological monitoring system monitors cardiorespiratory changes. Bispectral index monitor monitors depth of sedation/level of consciousness. Intra-oral camera monitors anatomical obstruction in the oral cavity. Electroencephalogram monitors sleep stage. Polysomnography (PSG) monitor monitors various physiological sleep related parameters. Video recording system monitors patient’s sleep behaviour (for unusual activities). Snoring recorder monitors snoring intensity and frequency.

The four key limitations of DISE data management are:

1. Anatomical data on dynamic upper airway obstruction from an endoscopic system and cardiorespiratory/clinical relevant parameters for clinical monitors are independently captured, independently viewed and independently stored.
2. A lack of correlation between upper airway obstruction parameters and cardiorespiratory/clinical relevant parameters in terms of severity, intensity and frequency of obstruction.
3. Aligning dynamic upper airway obstruction data from an endoscopic imaging system and cardiorespiratory/other clinical relevant data can be time consuming.
4. ENT surgeons and anaesthetists view two independent screens at different locations.

Three DISE DATA FUSION system solutions have been proposed in the literature:

In 1982, the first DATA FUSION system for capturing both anatomical and physiological data during sleep apnea for natural sleep was proposed [19]. The key advantage of this proposed system was capturing the upper airway dynamics and physiological parameters simultaneously in real time. However, the result of this captured data had not been reported and such technology is outdated and currently not available. In 2013, a customised video screen was proposed for fusing anatomical and physiological data during DISE [20]. This was the first study to demonstrate a DATA FUSION system that can potentially capture upper airway obstruction; physiological parameters from a PSG monitor and the pulse oximeter and BIS index from a BIS monitor. However, this is only achieved using a customised screen system. In 2017, DISE-PG, a technique for capturing and visualising anatomical and physiological data simultaneously in real time was developed [21]. DISE-PG provides an accurate comprehension of the upper airway obstructive dynamics and a non-obstructive breathing pattern. However, DISE-PG is limited to only DISE and polygraphic data.

The key objective of this study is to propose DISE DATA FUSION system, a new clinical technology technique for capturing, merging, displaying and storing upper airway obstruction data from an endoscopic imaging system and its associated cardiorespiratory parameters from an anaesthesiological monitoring system simultaneously in real time during DISE based on image fusion technique.

## Methods

### DISE DATA FUSION system

An endoscopic monitoring system (KARL STORZ image 1 hub 222010 20) was used to capture data on upper airway obstruction. An anaesthesiological monitoring system data (CARESCAPE Monitor B650) was used to capture cardiorespiratory parameters during upper airway obstruction. The DVI input of the endoscopic monitoring system was connected to DVI input (first input) of Picture-In-Picture Video Processor (MVP-100 Picture-In-Picture Video Processor) via a DVI-D to VGA converter, VGA cable and VGA to DVI converter. The DVI input of the anaesthesiological monitoring system monitoring system was connected to the VGA input (second input) of Picture-In-Picture Video Processor via a DVI to VGA converter and a VGA cable. The output of the Picture-In-Picture Video Processor was connected to the input of a video grabber (epiphan video DVI2USB 3.0) via a DVI to VGA converter and VGA to DVI cable. The output of the video grabber was connected to a laptop (Lenovo Y50) via a USB 3.0 b-Type connector cable.

### Clinical setup

**Fig. 1:**
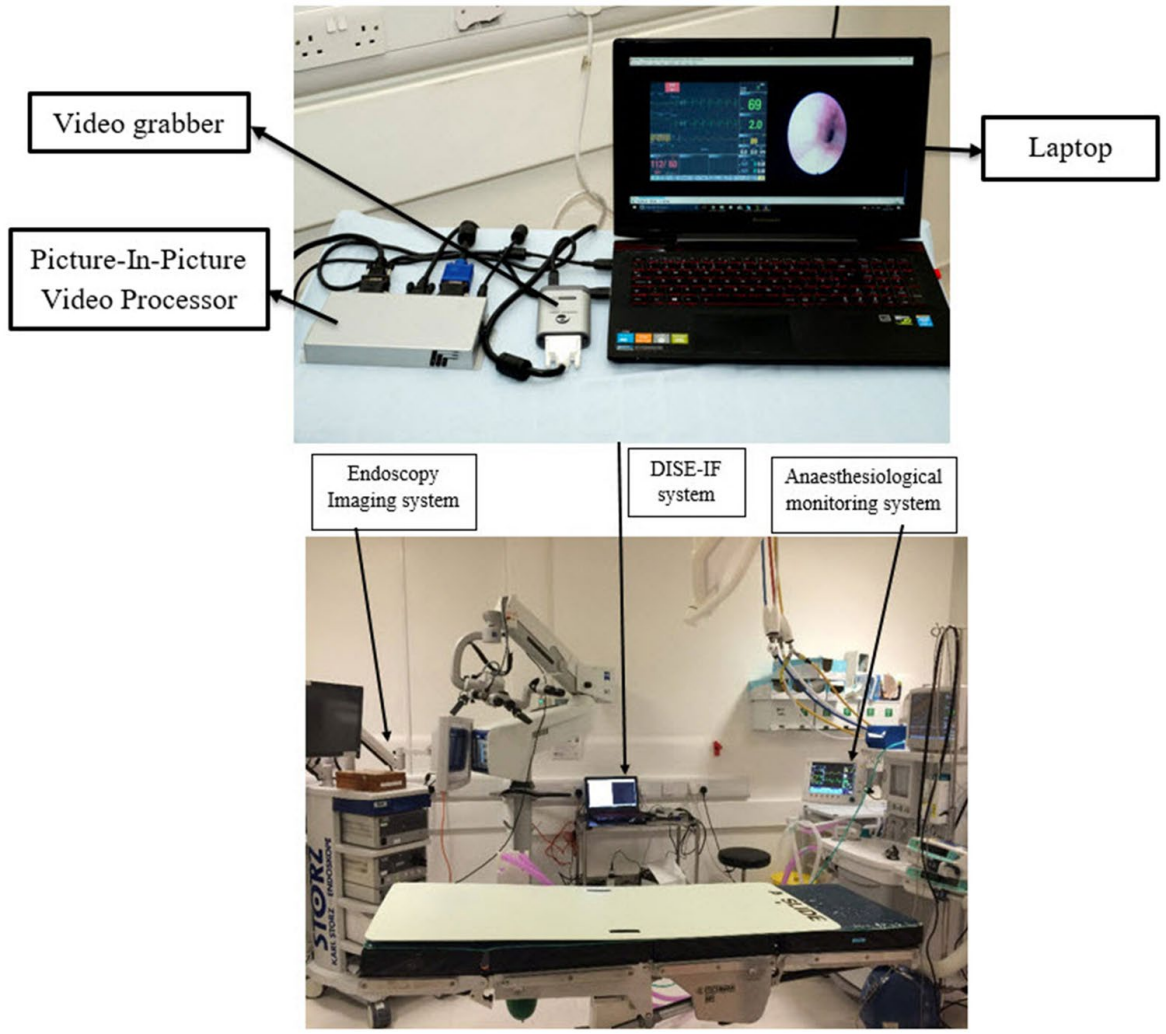
Clinical device setup

20 patients with complaints of sleep disordered breathing anonymously volunteered for DISE DATA FUSION system to be used during the DISE examination at the Royal National Throat, Nose, and Ear Hospital. All patients had previously undergone a sleep study and OPD evaluation [22]. All patients formally consented and no personal information about any patient was recorded. DISE was performed with midazolam (0.05 mg kg^−1^) and propofol (1.5 mg kg^− 1^). A DISE DATA FUSION system was used to capture, merge, visualise, and store dynamic upper airway anatomical changes from the endoscopic imaging system and cardiorespiratory changes from the anaesthesiological monitoring system simultaneously in real time. Figure 1 shows clinical device setup of DISE DATA FUSION system.

An assessment of snoring was made at the appropriate level of sedation and snoring by an expert assessor. The values of the oxygen saturation levels occurring during natural sleep, whilst having the sleep study, were used as indicators for appropriate timing of introducing a fibre-optic endoscope. The assessment is purposely not made on the onset of snoring but delayed until one cycle of apnea is followed by breakthrough and then repeated snoring occurs. It is important to note that initial sedation tends to lead to a deeper sleep than natural sleep. Also, this is helpful in allowing the passage of the fibre-optic endoscope without nasal irritation. The fibre-optic endoscope is held in place until one cycle of sleep disordered breathing had passed. The assessment occurs on the second and third cycle of snoring following apnoeic episodes and breakthroughs.

## Results

**Fig. 2:**
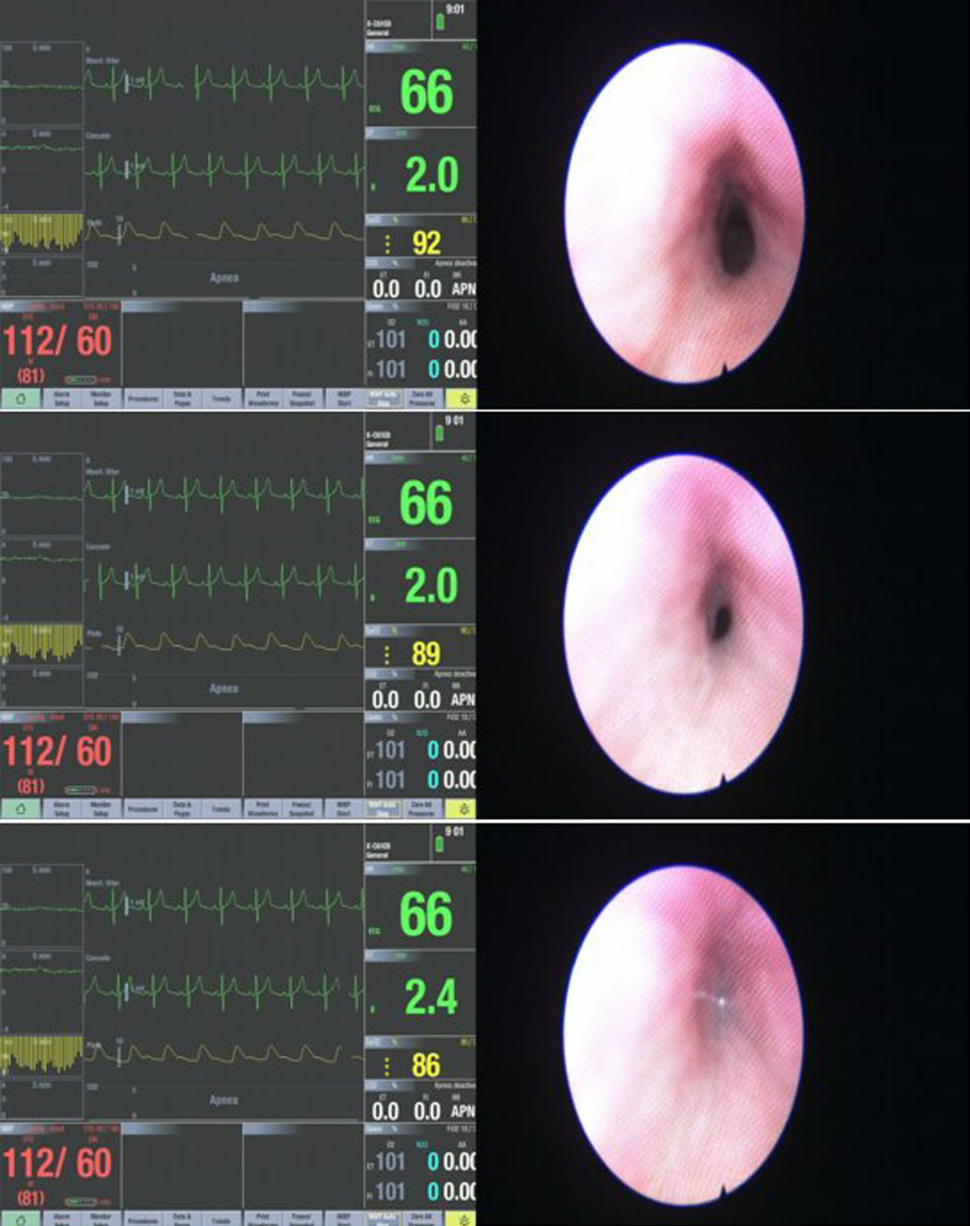
Sequence of the palatal obstruction

**Fig. 3:**
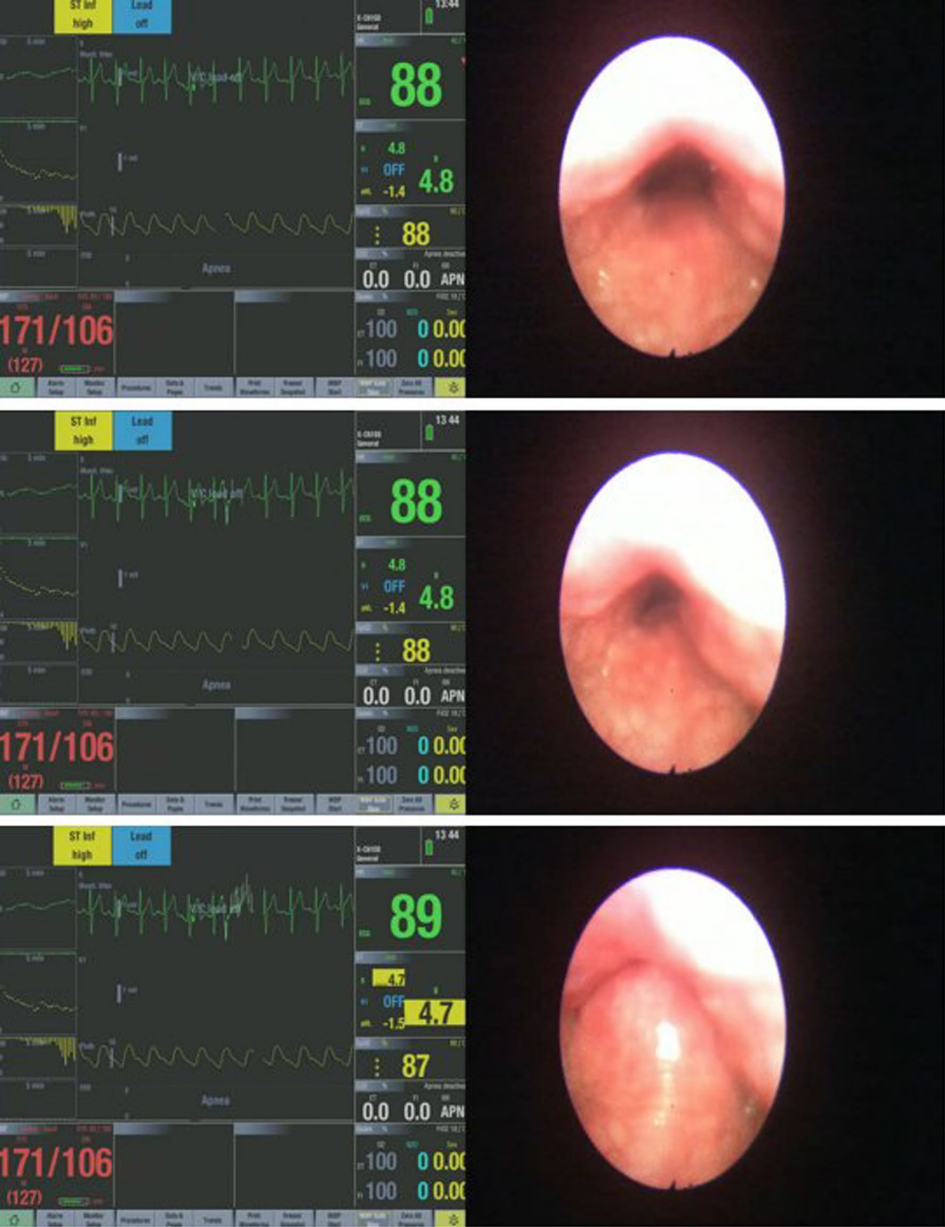
Different stages during a uvula-based upper airway obstruction

**Fig. 4:**
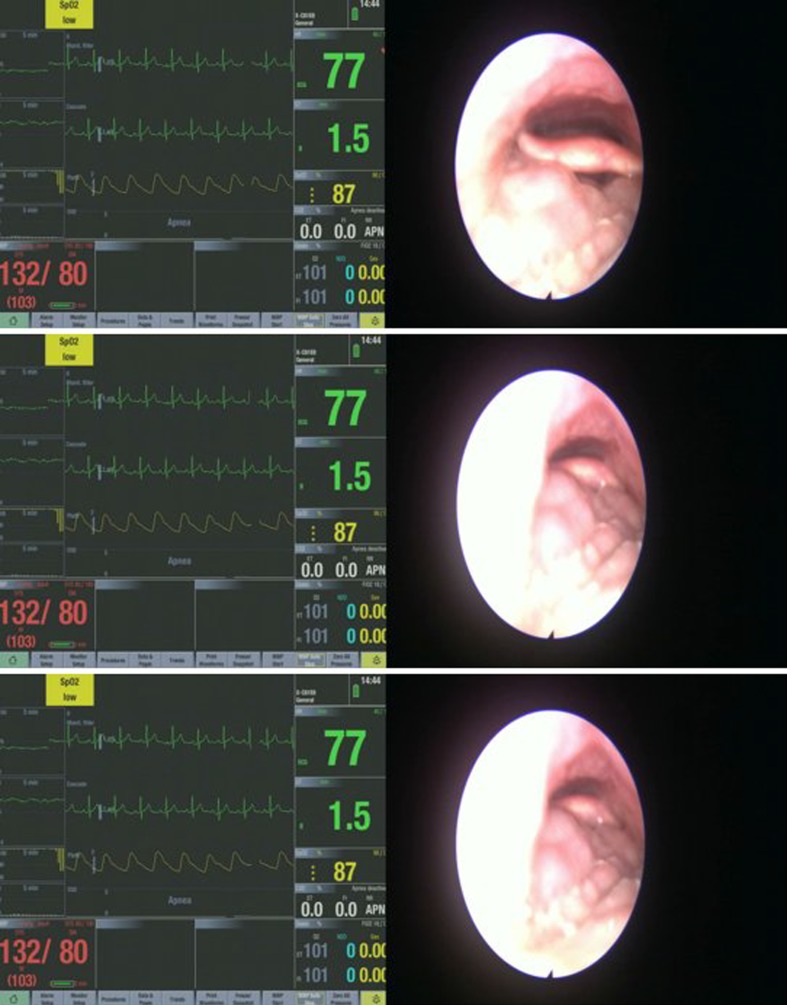
Different stages during a tongue-based obstruction

**Fig. 5:**
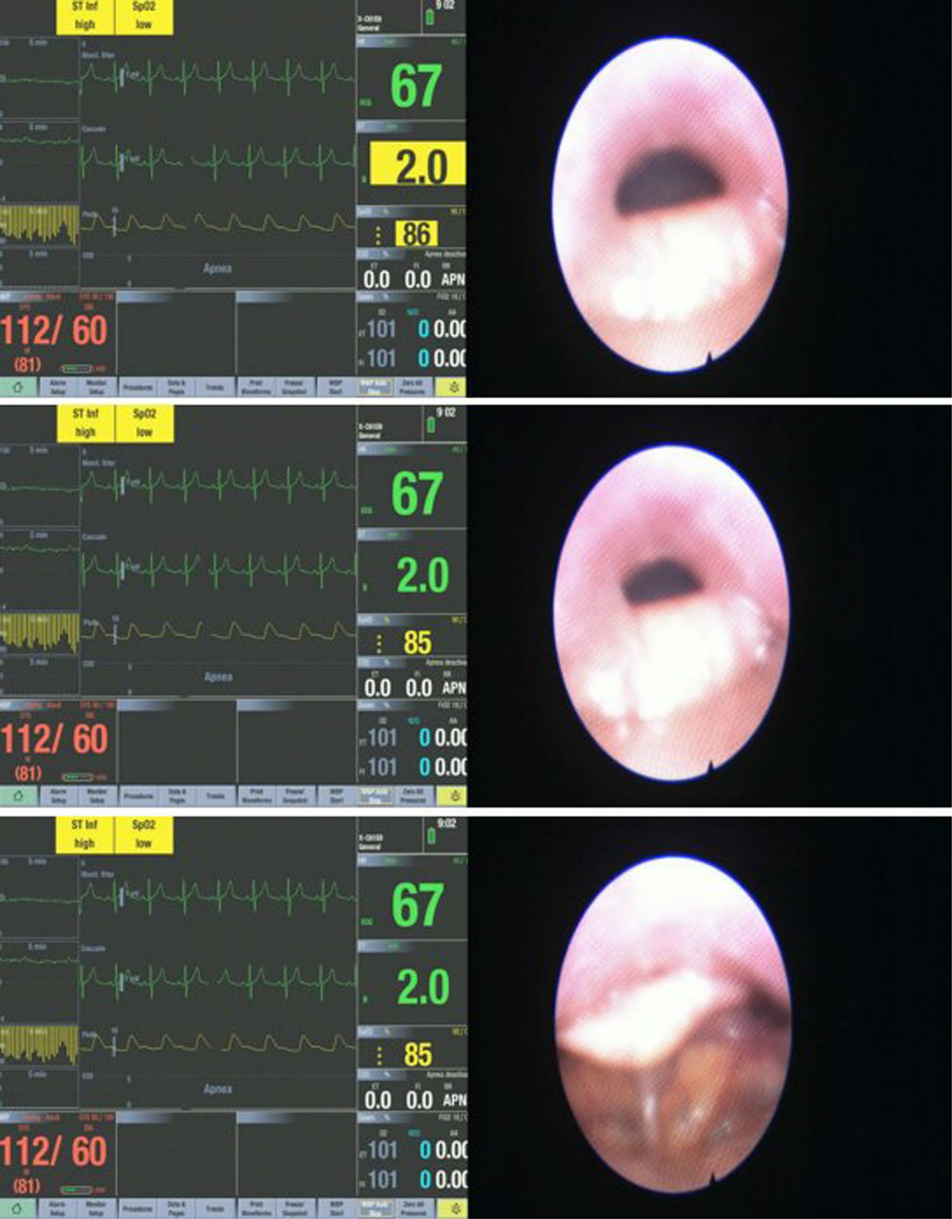
Different stages of an epiglottis trapdoor phenomenon

**Fig. 6:**
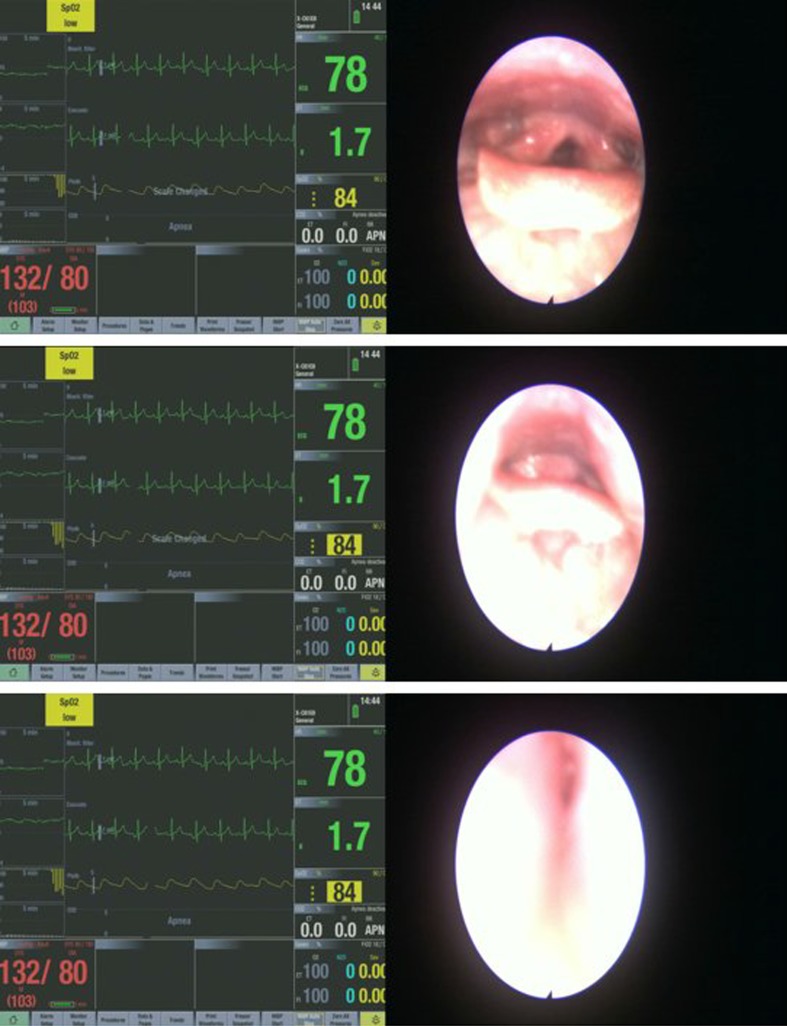
Different stages of the lateral pharyngeal wall upper airway obstruction

**Fig. 7:**
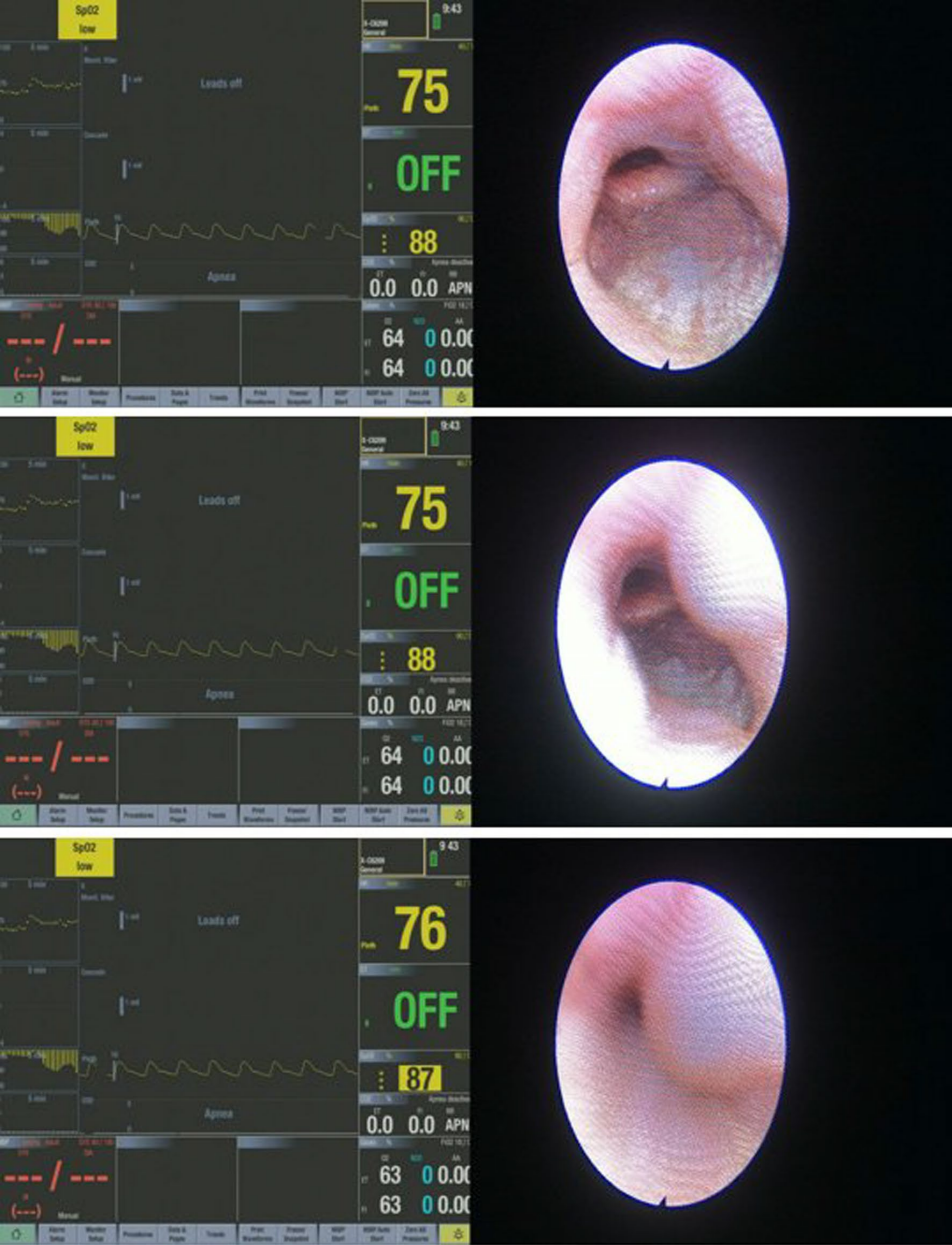
Tonsil-based upper airway obstruction

**Fig. 8:**
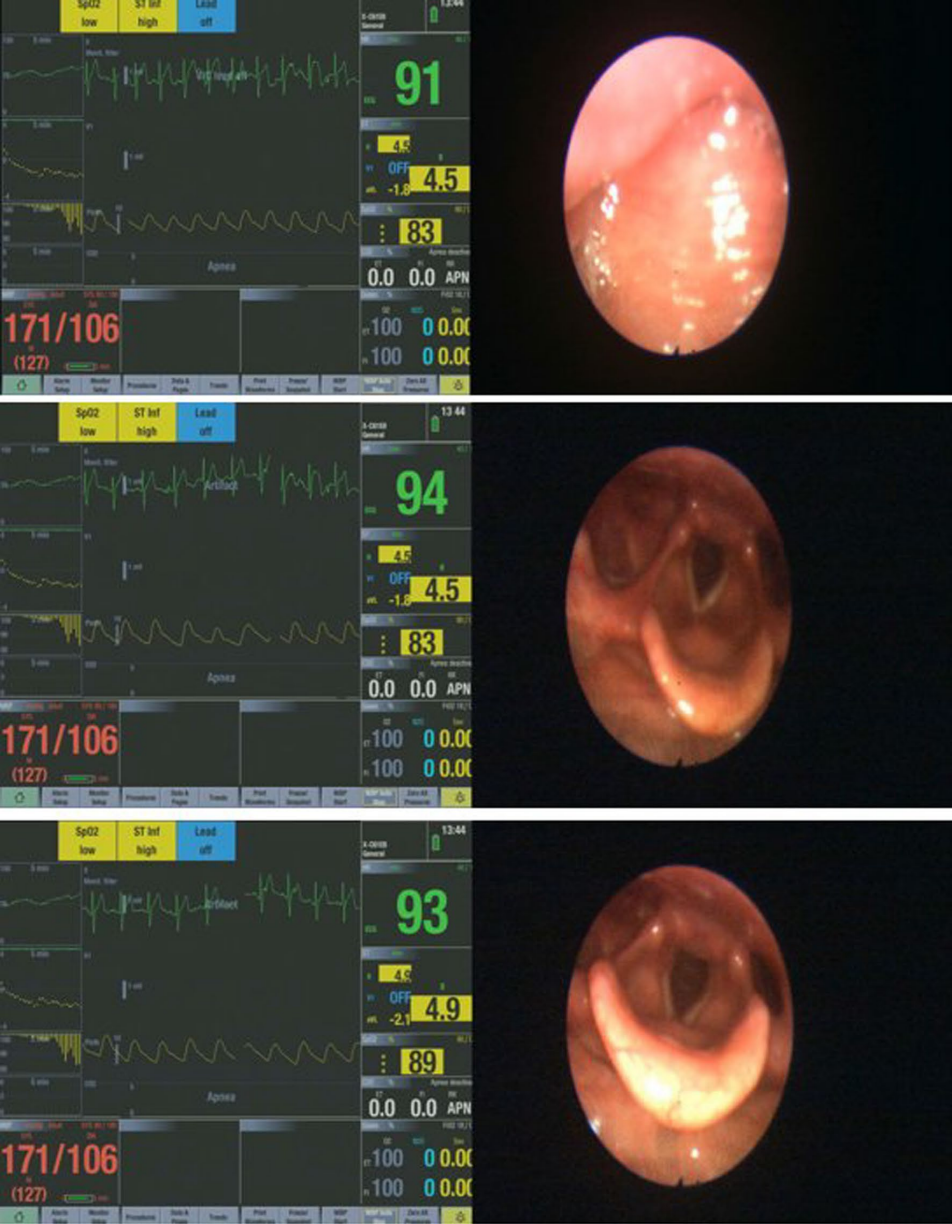
Effect of jaw thrust on an upper airway obstruction

The different upper airway obstruction patterns observed in the preliminary visualisation results of the DISE DATA FUSION system are shown in Figs. 2, 3, 4, 5, 6, 7 and 8.

Figure 2 shows a sequence of the palatal obstruction. The upper airway begins to collapse in a circumferential configuration. This results in an increase in negative pressure in the upper airway. In this example, the subject starts to desaturate at a blood oxygen level drop of 92%. As the apnea episode gets more severe, the negative pressure is increased. This causes a further collapse and a further desaturation of the subject until there is a total palatal obstruction. At this point, the maximum negative pressure occurs. The subject experiences a minimum blood oxygen saturation level at 86%. Worth noting in these images is that the oxygen saturation is 92% in the first image, where the lumen of the nasopharynx is fairly patent and as the respiratory cycle continues, the lumen continues to close more in less than a second and is then almost completely occluded in the third image reflected by the drop in oxygen levels of 89% and then 86%.

Figure 3 shows the different stages during a uvula-based upper airway obstruction. The obstruction configuration is in the anterior–posterior direction. There is no significant variation in the drop of the saturated blood oxygen level as the subject’s blood oxygen level dropped by only 1%. An elongated uvula can also lead to more severe vibration and flipping around during inspiration leading to a prolapse of the uvula into the nasopharynx during expiration. The vibration and the movement of the uvula can cause simple palatal snoring or lead to a severe apnea episode. It was noted that the blood pressure in this patient was high (uncontrolled hypertensive) and this fact was passed on to the primary care physician.

Figure 4 shows different stages during a tongue based obstruction. The tongue base moves back into the pharyngeal lumen onto the posterior pharyngeal wall in an anterior–posterior direction. The tongue base causes a complete obstruction. It is important to note that a similar obstruction can be caused by the posterior pharyngeal wall closing down onto the tongue base. The traction of the tongue posteriorly pushes the epiglottis downward and inward. This leads to obstruction in the pharyngeal cavity and in the hypopharynx causing a suction effect around the epiglottis.

Figure 5 shows different stages of an epiglottis trapdoor phenomenon. At the start of the epiglottis trapdoor, there is a notable gap between the epiglottis and the pharyngeal wall. As the epiglottis moves posteriorly, the severity of the obstruction increases until there is no gap between the epiglottis and the pharyngeal wall causing a total obstruction. In this example, the epiglottis trapdoor is caused entirely by the epiglottis moving in a posterior direction towards the pharyngeal wall with the structural integrity of a normal epiglottis. Another form of epiglottis trapdoor is caused by the anteroposterior prolapse of the pharyngeal wall [23]. This results in the folding of the epiglottis and a decreased structural rigidity of the epiglottis and its surrounding upper airway structure. An alternative airway configuration for the epiglottis trapdoor is a lateral folding or involution. A central vertical oriented crease of decreased rigidity of the epiglottis and the pharyngeal wall enables the epiglottis to fold and cause obstruction.

Figure 6 shows different stages of lateral pharyngeal wall upper airway obstruction. As the lateral pharyngeal wall obstructs, the pharyngeal wall starts to move laterally. As a further lateral movement occurs, it leads to a total blockage. In some cases, a total white screen is observed on the endoscopic screen. The pharyngeal wall obstruction is characterised by the movement of the lateral pharyngeal wall towards the centre of the airway. In this example, the obstruction occurred when the subject was already desaturated at a blood oxygen level of 84%. In some classification systems, the lateral pharyngeal wall serves as the only anatomical structure required for an oropharyngeal obstruction while both pharyngeal wall and tonsils are considered during an oropharyngeal obstruction [18].

Figure 7 shows different stages of a tonsil-based upper airway obstruction. At the beginning of the obstruction, the tip of the tonsils, tongue base, tip of the epiglottis, and lateral pharyngeal wall are visible. As the obstruction continues, the tonsils move laterally inward into the pharyngeal lumen towards the centre of gravity. This causes the tongue base and the lateral pharyngeal walls to move downward and inward sometimes causing an epiglottis retraction. This becomes a multisegmental upper airway obstruction. It has been shown that there is a strong correlation between the clinical tonsil grade and the obstructive tonsil volume in snoring adults and a significant correlation between the tonsil volume and the AHI index [24].

Figure 8 shows the effect of jaw thrust on an upper airway obstruction and represents patient from Fig. 3 with an uncontrollable hypertensive blood pressure. The jaw thrust was preceded by a total anteroposterior palatal obstruction at a saturated blood oxygen level of 83%. A jaw thrust manoeuvre is performed. The first notable observation was the oropharynx and the hypopharynx became visible. However, the oxygen saturation level remains unchanged. After a couple of seconds, the blood oxygen saturation level improved from 83 to 89%. The jaw thrust manoeuvre is used to simulate a mandibular advancement device (MAD) and check if the snoring and upper airway obstruction decreases. In this subject, it is clear that a mandibular advancement device could help the subject improve the upper airway obstruction. However, an improvement is not necessarily always possible.

## Discussion

This study proposes and presents preliminary results on DISE DATA FUSION System as a system for capturing, merging, displaying and storing upper airway obstruction from an endoscopic imaging system and its associated cardiorespiratory parameters/changes simultaneously in real time during DISE. This study presents eight discussion sections.

### Solution

A DISE DATA FUSION system provides a solution to the initial observed clinical problems by

1. Capturing, merging, visualising, and storing dynamic upper airway data from an endoscopic imaging system and cardiorespiratory data from the anaesthesiological monitoring system simultaneously in real time during DISE.
2. Allowing ENT surgeons and anaesthetists to simultaneously view one screen with both dynamic upper airway data from an endoscopic imaging system and cardiorespiratory data from the anaesthesiological monitoring system during DISE.
3. Providing aligned data after the end of the study: making the post-processing of data easier, less time consuming, and more manageable.
4. Providing datasets for finding and understanding how the upper airway obstruction parameters and patterns correlate with their corresponding cardiorespiratory parameters in terms of the obstruction severity and intensity.

### DISE multiple information management system

What is the best way of capturing, merging, displaying and storing multiple information during DISE? Borowiecki et al. proposed a multiple camera system for combining anatomical and physiological data during natural sleep [25]. The system proposed by Borowiecki et al. is technologically outdated. Furthermore, analysing 6–8 h of natural sleep data is time-consuming and not cost effective. Abdullah et al. proposed a customised screen system from capturing, merging and displaying upper airway obstruction, polysomnography data, bispectral index data and pulse oximetry data simultaneously data in real time during DISE [20]. The key limitation of the technique proposed by Abdullah et al. is dependent on a customised screen. At the time of this study, the customised screen was not available for purchase or research purposes. Hence, this affects clinical adoption, ease of use and availability of the customised screen system for DISE data fusion management. Gobbi et al. recently proposed DISE-PG technique for capturing, merging, displaying and storing upper airway obstruction and cardiorespiratory parameters simultaneously in real time during DISE [21]. The key limitation of DISE-PG technique is implementation dependency on technological solutions provided by Embla System Inc.

In the current study, DISE DATA FUSION system based on image fusion technique is proposed for capturing, merging, displaying and storing multiple information during DISE. As compared to the system proposed by Borowiecki et al., DISE DATA FUSION is currently technological feasible and implementable. As compared to the system proposed by Abdullah et al., DISE DATA FUSION system does not require a customised screen system for data management. Any monitor can be used for data visualisation when using DISE DATA FUSION system. As compared to DISE-PG technique, DISE DATA FUSION system is not reliant on any technological provider. DISE DATA FUSION system can be used on any clinical monitoring system during DISE.

### Information architecture for operative surgical decision

An important requirement for capturing, merging, displaying and storing multiple information during DISE is to improve DISE surgical decision-making process for optimal sleep surgery outcome. This raises the question: ‘what is the optimal information needed for optimal surgical decision?’ In this study, the information architecture is upper airway obstruction and its associated cardiorespiratory parameters (including heart rate, blood oxygen level and blood pressure). The information architecture proposed by Abdullah et al. is upper airway obstruction, polysomnography data, BIS data and pulse oximetry data (heart rate and blood oxygen level). The information architecture proposed by Gobbi et al. is upper airway obstruction data and cardiorespiratory data from polygraphic traces.

Upper airway obstruction data are the most critical information required for DISE-driven surgical decision-making process as it informs an ENT surgeon on the site of upper airway obstruction, obstruction configuration and severity of obstruction. BIS values inform an ENT surgeon on the state of consciousness or depth of sedation during observed upper airway obstruction. A BIS index score of 50–60 is recommended for surgical decision [26]. Blood oxygen level is the most used cardiorespiratory parameter in DISE surgical decision-making process as it informs an ENT surgeon on how desaturated a patient is during an upper airway obstruction. Sound information (snoring) relates the severity and frequency of apnoeic/obstructive events. In addition, snoring sound is used to determine when the endoscopy is inserted for DISE assessment: second cycle of snoring is preferred for insertion of endoscopy for DISE assessment. Polysomnography data are used to inform an ENT surgeon on sleep physiology of an obstructive event.

The ranking of secondary data associated with upper airway obstruction data remains subject to further research. Information required for optimal DISE surgical decision-making process includes upper airway obstruction data, snoring data, BIS value, cardiorespiratory data and polysomnography data.

### Information delivery and information management for post-operative functions

Efficient and effective information delivery of a DISE multiple information management system is important for post-evaluation data assessment, pre-operative surgical decision making process and research. Currently, multiple information on DISE information is delivered independently in most DISE assessment centres. This makes accessing important data for post-evaluation data assessment, pre-operative surgical decision process and research very challenging and time consuming. In most cases, it is impossible to replicate surgical decision made during DISE assessment because multiple information visualised during DISE is not stored and captured simultaneously. The customised screen proposed by Abdullah et al. does not provide a storage management for reproducing data and information delivery. DISE-PG technique delivers information efficiently on a laptop (or similar computing device). However, visualisation of delivered information by DISE-PG technique depends on availability of Embla software. This raises barriers to information delivery. One key advantage of DISE DATA FUSION system is that information is delivered in a video format. Hence, any laptop or computing device with a video player will be able to access captured information by DISE DATA FUSION system. It is important to note that a video player is a standard software package on a laptop/computer. Thus, this makes post-operative information management/activities with DISE DATA FUSION system user friendly, efficient, effective and not technology dependent. Furthermore, data produced by DISE DATA FUSION system can be easily transferred via secured video sharing platform.

### Frame rate

In the customised screen system proposed by Abdullah et al., sleep stages and BIS values were recorded every 30 s (0.033 frame per second) and bispectral analysis values were recorded every 10 s (0.1 frame per second). It is not stated the overall information frame rate. The video frame rate of DISE-PG technique is 15 frames per second with polygraphic traces much higher. The frame rate of information captured by DISE DATA FUSION system is 25 frames per second with a potential maximum of 50 frames per second. Thus, DISE DATA FUSION system currently provides the highest information frame rate.

### Device abstraction

From an innovation viewpoint, it is important for the system capturing, merging, display and storing information to be device independent. The customised screen system and DISE-PG system are dependent on monitoring system. Hence, this limits usability and clinical adoption. DISE DATA FUSION system uses image fusion technique which means any monitoring system with a video output or a display screen can be used as an information source. Hence, any monitoring system can be abstracted/represented as a system with a video output and/or a display screen (independent of the manufacturer of the monitoring system). Hence, it is valid to assume that DISE DATA FUSION system will work on any monitoring system that has a video output and/or a display screen. This increases the spectrum of devices that can be used with DISE DATA FUSION system. Thus, it increases clinical implementation, clinical usabilty and clinical adoption of DISE DATA FUSION System.

### Flexible information source

One advantage of DISE DATA FUSION system is flexibility of information source. Due to the fact that DISE DATA FUSION system uses image fusion technique, any two information sources can be used based on user preferences. It is advisable that one of the information sources is an endoscopic imaging system as it contains visual information on upper airway obstruction. For the customised screen system, the information source is fixed to endoscopic imaging system, BIS monitor, PSG monitor and pulse oximeter. For the DISE-PG technique, information source is fixed to endoscopic imaging system and polygraphic traces.

### Limitations

The key limitation of DISE DATA FUSION system is reliance on video output or a display screen. However, most monitoring systems used for DISE assessments have a video output or a monitoring screen for ENT surgeons to visualise data. Most monitoring systems without a video output or a display screen tend to have a software package for visualising captured data. Hence, there is a very small possibility/scope where DISE DATA FUSION system will not work.

Another limitation of the current version of DISE DATA FUSION system is it can only capture information from two independent sources. It is important to note that DISE-PG technique captures information from only two information sources. Only the customised screen system captures information from four different information sources. Hence, the customised screen system currently has the highest information source capture rate. Further research on increasing the number of information sources of DISE DATA FUSION from 2 information sources to 4 information sources is ongoing.

## Conclusion

DISE DATA FUSION system provides a better way of capturing, merging, visualising and storing anatomical data from a fibre-optic endoscope and its associated cardiorespiratory parameters from an anaesthesiological monitoring system simultaneously in real time during DISE. Firstly, DISE DATA FUSION system allows ENT surgeons and anaesthetists to simultaneously visualise both anatomical and physiological parameters on one screen during DISE. Secondly, DISE DATA FUSION system creates a composite data structure consisting of an anatomical image, blood oxygen level, pulse rate, blood pressure, and timestamp for every obstructive episode. Thirdly, DISE DATA FUSION system provides a pathway for further studies in gaining a deeper understanding of how the dynamic upper airway relates to drug sedation and physiological changes. A set-up of this nature allows better understanding of anatomical abnormality with the relevant change in physiological parameters. Lastly, DISE DATA FUSION system enhances the understanding of the impact of the anatomical severity due to the simultaneous display of the cardiovascular parameters at that specific time of anatomical obstruction for optimising the surgical decision based on DISE.

## Electronic supplementary material

Below is the link to the electronic supplementary material.

Supplementary material 1 (MP4 78264 KB)

Supplementary material 2 (MP4 17579 KB)

Supplementary material 3 (MP4 26987 KB)
